# Assessment of Knowledge, Attitude, and Practices Regarding Self-medication for Acne Among Medical Students

**DOI:** 10.7759/cureus.5510

**Published:** 2019-08-28

**Authors:** Ahsan Tameez-ud-din, Ifrah J Malik, Awais A Bhatti, Asim Tameez Ud Din, Abdullah Sadiq, Muhammad T Khan, Noman A Chaudhary, Daneyal Arshad

**Affiliations:** 1 Internal Medicine, Rawalpindi Medical University, Rawalpindi, PAK; 2 Surgery, Rawalpindi Medical University, Rawalpindi, PAK

**Keywords:** acne, self-medication, medical students

## Abstract

Introduction

Acne is one of the most common skin conditions worldwide. Self-medication for acne is a fairly common practice among medical students. The objective of our study was to identify the prevalence of self-medication and to assess its knowledge, attitude, and practices among medical students.

Materials and methods

This descriptive cross-sectional study was conducted at Rawalpindi Medical University, Rawalpindi, Pakistan, from January 2019 to June 2019. Data were collected by using the convenient sampling technique. Students were asked to fill a semi-structured questionnaire. Students of all the medical years studying in our university were included in the study. Data were entered and analyzed using the Statistical Package for Social Sciences (SPSS) version 23.0 (IBM Corp., Armonk, US).

Results

Out of 349 students, 244 (69.9%) suffered from acne and self-medication was practiced by 123 (50.4%) acne sufferers. The practice of self-medication was significantly higher in students having acne lesions on the face (52.2%). The most common source of information was reported to be acquaintances (55.8%). Most of the students had knowledge of the dosage of drugs (46.3%) and precautions for their use (41.5%). Sixty-three percent of the students were of the opinion that self-medication is part of self-care. Most of the students read the expiration date on the drug label (88.6%).

Conclusion

Acne is a highly prevalent condition among medical students and the practice of self-medication among acne sufferers is high. The practice of self-medication and visits to dermatologists were both significantly more common in the students with lesions on the face. The knowledge of students regarding self-medication of acne was not adequate.

## Introduction

Acne is one of the most common skin conditions affecting teenagers of both sexes worldwide [1]. It is an inflammatory condition that results from an underlying pathology involving sebaceous glands of the skin. There are different morphological types of pimples or acne such as whiteheads, blackheads, cysts, nodules, and pustules [2]. Untreated acne may lead to scarring. The face is one of the most common sites for acne lesions, which results in considerable distress to the patient, usually out of proportion to the disease severity. The psychological and social impacts of acne have been the driving forces behind intensive efforts to find a suitable cure even for mild cases [3].

Self-medication is defined by the World Health Organization (WHO) as the “use of medicinal products by the consumer to treat self-recognized disorders or symptoms or the intermittent or continued use of medication prescribed by a physician for chronic or recurring diseases or symptoms.” Self-medication is a fairly common practice among medical students due to a variety of factors such as ease of availability, exposure of medical settings, and pharmacological knowledge [4]. Even though the WHO advocates self-medication for the treatment of minor ailments, it does caution against its pitfalls such as adverse side effects and the emerging resistance among pathogens [5].

The aim of this study was to aid the decision-makers in incorporating basic dermatological knowledge in the medical curriculum to avoid risky self-medication behaviors and to improve the clinical acumen of future doctors. The objectives of this study were to find the prevalence of acne vulgaris in medical students of Rawalpindi Medical University and to assess the knowledge, attitude, and practices regarding self-medication for acne vulgaris in medical students.

## Materials and methods

This descriptive cross-sectional study was conducted at Rawalpindi Medical University, Rawalpindi, Pakistan, from January 2019 to June 2019. Students of all the medical years studying in our university were included in the study. Data were collected by using the convenient sampling technique. Students were asked to fill a semi-structured questionnaire. Incompletely filled questionnaires were excluded from the final results. Most of the questions were taken from a study by Karamata et al. [6]. The questionnaire had two parts, the first consisted of a demographic portion and questions about the site of acne, the pattern of self-medication, and the source of information. The second part of the questionnaire comprised questions regarding knowledge, attitude, and practices of self-medication. Knowledge was assessed by six questions, including knowledge about dose, mechanism of action, precautions for use, complications, side effects, and contraindications of the drugs being used. The sections of attitude and practices had four and three questions, respectively. The results were reported as frequencies and percentages. Data were entered and analyzed using the Statistical Package for Social Sciences (SPSS) version 23.0 (IBM Corp, Armonk, US). Chi-square tests were applied for qualitative variables. A p-value of less than 0.05 was considered significant.

## Results

Out of 349 students, 104 (29.8%) were males, and 245 (70.2%) were females. The mean age of the students was 21.2 ± 1.9 years. Seventy-two (20.6%) students were from the first year, 40 (11.5%) were from the second year, 52 (14.9%) were from the third year, 62 (17.8%) were from the fourth year, and 112 (32.1%) students belonged to the final year. The medical year was not reported by 11 (3.2%) participants.

The prevalence of acne among medical students was 69.9% (n = 244). The sites of acne lesions are shown in Table 1. Six (2.5%) students did not mention the site of acne lesions.

**Table 1:** Sites of acne lesions

Region	Frequency (%)
Face	168 (68.9%)
Face and back	36 (14.8%)
Face, chest, and back	20 (8.2%)
Back	8 (3.3%)
Face and chest	4 (1.6%)
Chest	1 (0.4%)
Chest and back	1 (0.4%)

Out of 244 students suffering from acne, 123 (50.4%) practiced self-medication. The practice of self-medication was significantly higher in students having acne lesions on the face (52.2%), whereas only 10% of students with acne involving sites other than the face practiced self-medication (p = 0.009). Dermatologist's consultation was sought by 134 (54.9%) students suffering from acne. Students with acne lesions involving the face consulted a dermatologist significantly more often (57.7%) than those with lesions on sites other than the face (20%, p = 0.019). Seventy-three (29.9%) students suffering from acne had a doctor in the family. Three (1.2%) students did not respond to this question. Self-medication was significantly more frequent in students with a doctor in the family (61.6%, p = 0.03).

Out of the 123 students who practiced self-medication, eight (6.5%) used only oral medications while solely topical drugs were applied by 73 (59.3%) students. Both oral and topical medications were used by 38 (30.9%) students. Four (3.3%) students did not mention the mode of drug administration.

Allopathy was the most common type of medication used by students (n = 75, 47.8%), followed by home-made remedies (n = 52, 33.1%). This is shown in Table 2.

**Table 2:** Type of medication

Type of medication	Frequency (%)
Allopathy	75 (47.8%)
Home-made remedies	52 (33.1%)
Homeopathy	14 (8.9%)
Ayurvedic medication	7 (4.4%)
Others (cosmetic products, chemical peels)	9 (5.7%)

The most common reason for self-medication was the mild nature of the disease (n = 49, 34.3%), followed by the easy availability of the drugs (n = 43, 30.1%). This is shown in Table 3.

**Table 3:** Reason for self-medication

Reason for self-medication	Frequency (%)
Mildness of disease	49 (34.3%)
Easy availability	43 (30.1%)
Know treatment from the previous prescription	20 (14%)
Lack of time	16 (11.2%)
Pharmacological knowledge	8 (5.6%)
Did not want to involve faculty	5 (3.5%)
The embarrassment of discussing symptoms	2 (1.4%)

Acquaintances were the main source of information for most of the students (n = 77, 55.8%), followed by the prescription issued to others (n = 20, 14.5%). This is shown in Table 4.

**Table 4:** Source of information

Source of information	Frequency (%)
Acquaintances	77 (55.8%)
Prescription issued to others	20 (14.5%)
Self-decision	13 (9.4%)
Drug ads	6 (4.3%)
Books	6 (4.3%)
Others (Internet, seminars, lectures)	16 (11.6%)

Most of the students knew the dose of the drug they were using (n = 57, 46.3%). Only 27 (22%) of the students knew about the contraindications of the drugs, as shown in Table 5. Three (2.4%) participants did not answer the knowledge section of the questionnaire.

**Table 5:** Knowledge of self-medication

Knowledge	Yes n (%)	No n (%)
Dose of drug	57 (46.3%)	63 (51.2%)
Mechanism of action	39 (31.7%)	81 (65.9%)
Adverse effects	38 (30.9%)	82 (66.7%)
Precautions for use	51 (41.5%)	69 (56.1%)
Complications	36 (29.3%)	83 (67.5%)
Contraindications	27 (22%)	93 (75.6%)

Eighty (65%) students felt that their condition improved after self-medication while four (3.3%) thought that the acne lesions worsened with the self-administration of drugs. Thirty-three students (26.8%) felt that self-medication did not affect the outcome of their acne lesions. Six (4.9%) students did not give a satisfactory answer to this question. The attitude of students towards self-medication is shown in Table 6. One (0.8%) student did not answer the first three questions while two (1.6%) students did not respond to the last question shown in the following table.

**Table 6:** Attitude towards self-medication

Attitude	Yes n (%)	No n (%)
Self-medication is part of self-care	77 (62.6%)	45 (36.6%)
Advise self-medication to friends/family	61 (49.6%)	61 (49.6%)
Is dermatologist’s consultation important for acne?	110 (89.4%)	12 (9.8%)
Is follow-up for acne important?	111 (90.2%)	10 (8.1%)

The practices of medical students regarding self-medication are shown in Table 7. One (0.8%) student did not respond to the first question while two (1.6%) students did not answer the last two questions as shown in Table 7.

**Table 7:** Practices regarding self-medication

Practice	Yes n (%)	No n (%)
Do you read instructions on the drug label?	88 (71.5%)	34 (27.6%)
Do you read the expiration date of the drug?	109 (88.6%)	12 (9.8%)
Is the medication always available at home/hostel?	54 (43.9%)	67 (54.5%)

## Discussion

Acne is a very common skin condition affecting teenagers and adolescents worldwide. The prevalence of acne in our setup was found to be 69.9%, which was consistent with the findings of Talanikar et al. [7]. Comparably, a study conducted in Karachi showed a prevalence of 55.9% among the students [8]. Self-medication for acne is a fairly common practice among medical students. According to our findings, 50.4% of the students suffering from acne practiced self-medication, which was on par with the findings of Corey et al. [9]. Similarly, Karamata et al. reported self-medication in 59.2% of acne sufferers which was close to our findings [6]. According to Raiker et al., a higher number of medical students (77.4%) practiced self-medication [1].

The face was the most commonly involved site of acne in our study, with 68.9% of lesions involving this area of the body. Alanazi et al. also reported the presence of acne lesions on the face in the majority of secondary school students [10]. The practice of self-medication was significantly more common in students having lesions on the face. Moreover, students with facial lesions also seek dermatologist’s consultation much more frequently than those with lesions on other body parts. The face is one of the most important parts of the human body from a cosmetic point of view and any visible lesion causes considerable distress to the patient. Further studies are required to clarify the relationship between the practice of self-medication and the site of acne lesions to improve the existing strategies for acne management. Similar comparisons have been made between gender and self-medication in some studies [6,11].

The most common reason for self-medication in our study was the mildness of the condition (34.3%), followed closely by easy availability (30.1%). The mild nature of the disease has been reported as the most common reason in many studies [6-7]. Acquaintances (seniors/friends/members of the family) were the most frequently cited source of information in our study (55.8%). Friends/seniors were also reported as the most common information source by Patil et al. [12]. The medical school curriculum is designed to give the students a thorough experience of the clinical settings, which results in a special bond of trust and friendship between batch mates and their seniors. For this reason, seniors and friends may influence medical students the most in their decision of self-medication.

Sixty-three percent of students in our study were of the opinion that self-medication is a component of self-care, which was close to the findings of Karamata et al. [6]. Around 50% of the students had no reservations about recommending the medication to their friends and family, similar to the results of Raikar et al. [1]. According to our findings, 89.4% of the students thought that the dermatologist's consultation was important for the treatment of acne. A study by Zafar et al. also reported similar findings [13]. The knowledge of the pharmacological properties and the effects of drugs used was less than 50%, which is alarmingly low as a lack of adequate knowledge may lead to severe and undesirable side-effects. Most of the students in our study used allopathic medication (47.8%) while homemade preparations were used by 33.1% of students. This was consistent with the findings of Kumar et al. [14]. The topical route of administration was most common in our study. Topical drugs were also the most commonly used drugs according to the findings of Karamata et al. [6]. The topical route is considered one of the safest routes of administration and the availability of a large number of over-the-counter topical drugs may make it a convenient choice for medical students.

The majority of the students in our study read the instructions and the expiration date on the drug label. While this is an encouraging statistic and reflects the cautious mindset of medical students regarding the use of medications, it does not address the problem of overuse of certain drugs, which may result in dangerous side-effects like drug resistance [15]. Self-medication is an emerging trend among medical students and effective measures are needed to curb the dangerous side-effects of self-medication.

There were some limitations to our study. Poor knowledge about the clinical definition of acne might have resulted in a slightly less than expected prevalence of acne and its self-medication. Some of the questionnaires had to be discarded due to inadequate responses.

## Conclusions

Acne is a highly prevalent condition among the medical students and the practice of self-medication among acne sufferers is high. The mildness of disease was the most commonly cited reason for self-medication. The practice of self-medication and the visits to dermatologists were both more common in the students with lesions on the face. The knowledge and attitude of medical students regarding the self-medication of acne mandate improvement in the existing dermatology curriculum. Awareness of the properties, side-effects, and the appropriate dosing schedule of the drugs may help control the emerging epidemic of drug resistance.
